# NPR-B natriuretic peptide receptors in human corneal epithelium: mRNA, immunohistochemistochemical, protein, and biochemical pharmacology studies

**Published:** 2010-07-07

**Authors:** Parvaneh Katoli, Najam A. Sharif, Anupam Sule, Slobodan D. Dimitrijevich

**Affiliations:** 1 Pharmaceutical Research, Alcon Research, Ltd., Fort Worth, TX; 2 Department of Integrative Physiology, University of North Texas Health Science Center, Fort Worth, TX

## Abstract

**Purpose:**

To demonstrate the presence of natriuretic peptide receptors (NPRs) in primary human corneal epithelial cells (*p*-CEPI), SV40-immortalized CEPI cells (CEPI-17-CL4) and in human corneal epithelium, and to define the pharmacology of natriuretic peptide (NP)-induced cGMP accumulation.

**Methods:**

NPR presence was shown by RT–PCR, western blot analysis, and indirect immunofluoresence. cGMP accumulation was determined using an enzyme immunoassay.

**Results:**

*p*-CEPI and CEPI-17-CL4 cells expressed mRNAs for *NPR-A* and *NPR-B*. Proteins for both NPRs were present in these cells and in human corneal epithelium. C-type NP (CNP), atrial NP (ANP) and brain NP (BNP) stimulated the accumulation of cGMP in a concentration-dependent manner in *p*-CEPI cells (potency; EC_50s_): CNP (1–53 amino acids) EC_50_=24±5 nM; CNP fragment (32–53 amino acids) EC_50_=51±8 nM; ANP (1–28 amino acids) EC_50_=>10 µM; BNP (32 amino acids) EC_50_>10 µM (all n=3–4). While the NPs were generally more potent in the CEPI-17-CL4 cells than in *p*-CEPI cells (n=4–9; p<0.01), the rank order of potency of the peptides was essentially the same in both cell types. Effects of CNP fragment in *p*-CEPI and CEPI-17-CL4 cells were potently blocked by HS-142–1, an NPR-B receptor subtype-selective antagonist (K_i_=0.25±0.05 µM in CEPI-CL4–17; K_i_=0.44±0.09 µM in *p*-CEPIs; n=6–7) but less so by an NPR-A receptor antagonist, isatin (K_i_=5.3–7.8 µM, n=3–7).

**Conclusions:**

Our studies showed the presence of NPR-A and NPR-B (mRNAs and protein) in *p*-CEPI and CEPI-17-CL4 cells and in human corneal epithelial tissue. However, detailed pharmacological studies revealed NPR-B to be the predominant functionally active receptor in both cell-types whose activation leads to the generation of cGMP. While the physiologic role(s) of the NP system in corneal function remains to be delineated, our multidisciplinary findings pave the way for such future investigations.

## Introduction

Atrial natriuretic peptide (ANP) [1] and brain natriuretic peptide (BNP) [2,3] activate cytoplasmic membrane-spanning receptors which themselves are guanylyl cyclases (GCs) [4-7]. Three sub-types of receptors (NPR-A, NPR-B, NPR-C) have been discovered so far that interact with the natriuretic peptides (NPs) [4-7]. While NPR-A and NPR-B mediate the effect of the NPs to generate cGMP, NPR-C is a so-called “clearance receptor” that apparently is not coupled to an effector system and is involved in eliminating NPs when the concentrations of the latter exceed a certain level [4-7]. While ANP and BNP selectively activate NPR-A, the C-type natriuretic peptide (CNP) binds selectively and with high affinity and stimulates NPR-B [4-8]. NPs have many diverse physiologic functions in vascularized tissues including natriuresis, diuresis, vasorelaxation, hormone secretion, and modulation of neural transmission and Ca^2+^-channel activity [4-7]. Furthermore, NPs can affect tissue contractility, and cell proliferation and differentiation via the cGMP that is generated upon NPR activation [5,9-11].

Overall, the roles of NPs in ocular functions are poorly defined especially in the avascular corneal epithelium. However, the presence of immunoreactive ANP has been reported in the anterior uvea, retina, aqueous humor, ciliary body, and lacrimal gland [12-15]. NPR-A has been observed on the epithelial side of the ciliary body [16] and in the retina [17], while NPR-B has been found in the trabecular meshwork (TM), ciliary muscle (CM), and in non-pigmented ciliary epithelial cell [18]. Topical or intracameral administration of NPs in rabbits causes a reduction in intraocular pressure (IOP) [19,20].

The corneal epithelium and the tear film that covers it represent important barriers to protect the eye from airborne pathogens, allergens, u.v. light, and chemicals [21]. A clear, undamaged cornea is of course also important to permit light entry through to the visual axis for proper vision. Thus, any damage to the corneal epithelial cells (CEPIs) caused by the aforementioned elements can result in inflammation, perforation, neovascularization, fibrosis, and scarring that can seriously reduce visual acuity and cause partial or complete blindness [21]. Therefore, it is important to better understand the physiology, pharmacology and pathology of the corneal surface and the resident CEPIs. Relative to NPs and the cornea, the locally released and/or lacrimal gland-derived NPs may influence the CEPIs upon their arrival in the tear film. Accordingly, while NPR-C, the clearance receptor, was found on the bovine corneal endothelium [22], a GC-coupled NPR-B was identified in total homogenates of whole bovine corneas where CNP was twice as potent at stimulating cGMP production as ANP [23]. However, the cell-type(s) responsive to the NPs in the whole corneal tissue homogenates was not elucidated [23]. Likewise, even though ANP inhibited the epidermal growth factor-induced cell proliferation in rabbit corneal epithelial cell cultures via an NPR-A [24], unfortunately other NP agonists (e.g., CNP) were not used by these investigators to define the pharmacological specificity of the NPRs present in the rabbit CEPI cells [24]. Consequently, there is poor understanding about the NPR system in the cornea, especially that of humans, and especially that of the CEPIs. The fact that the CEPIs are probably important in mediating the effects of locally produced NPs and/or those released from the lacrimal gland [17] underscores the need to better define the presence of and to characterize the pharmacology of functionally active NPRs on the human CEPIs. The results from such studies may reveal important new means to positively influence corneal epithelial health and may lead to discovery of new therapeutic agents to promote corneal wound healing and/or treat dry eye and related disorders. Therefore, the aims of our studies were to demonstrate the presence of NPRs in the human corneal epithelium, primary human corneal epithelial (*p*-CEPI) cells, and in a human corneal epithelial cell-line (CEPI-17-CL4) [25-28] at the mRNA and protein levels. We further characterized, functionally and pharmacologically, the predominant NPR present in *p*-CEPI and CEPI-17-CL4 cells using a range of agonists and antagonists and measurement of cGMP production.

## Methods

### Materials

Sources of materials, reagents, equipment used were as follows: NPs from American peptide (Vista, CA) or Bachem (San Carlos, CA); various other compounds from either Sigma-Aldrich (St. Louis, MO), Tocris (Ellisville, MO) or Biomol (Plymouth Meeting, PA); HS-142–1 was a generous gift from Kyowa Hakko Co. (Tokyo, Japan); RT–PCR materials from InVitrogen (San Diego, CA); Gel electrophoresis materials from Gibco BRL (Carlsbad, CA) and New England Biolabs (Ipswich, MA); Immunocytochemicals from Molecular Probes (Portland, OR), Calbiochem (La Jolla, CA), and Fisher Scientific (Pittsburgh, PA); NPR antibodies from Abcam (Cambridge, UK); western blot materials from Pierce (Rockford, IL), Sigma-Aldrich or Amersham Biosciences (Buckinghamshire, UK).; Enzyme immunoassay kits from Amersham (Piscataway, NJ); Cell culture reagents and materials from BD Biosciences (San Jose, CA), Cascade Biologicals (Portland, OR), Gibco BRL, or Sigma-Aldrich. All tissues were obtained in compliance with good clinical practices with informed consent under institutional review board regulations and with the tenets of the Declaration of Helsinki.

### Cell Culture

#### Corneal Epithelial Cells (p-CEPI and CEPI-17-CL4)

Epithelial sheets were obtained from Eye Bank corneas from ocular pathogen-free donors (aged 40–86 years) and the primary cells cultured as previously described [25-28]. Briefly, after incubation of corneas at 4 °C for 48 h in dispase (diluted with calcium free EpiLife® medium to 12 Units/ml), the epithelial sheets were removed from the stromas, dissociated into single cell suspension, and plated on to murine-collagen-IV-coated tissue culture flasks (75 cm^2^). The cells were cultured in EpiLife^® ^(Cascade Biologics Invitrogen, Carlsbad, CA), a serum free defined media containing human corneal growth supplement, to 80% confluence. The cells were sub-cultured by harvesting with trypsin / EDTA, treatment with trypsin inhibitor and plating into murine-collagen-IV-coated multi-well plates.

The generation and extensive characterization of simian virus (SV)-40 immortalized human *p*-CEPI cells (clone CEPI-17-CL4) has been previously documented [25-28]. CEPI-17-CL4 cells (passages 58–158) were cultured and plated into multi-well plates as described above for *p*-CEPI.

### PCR

RNA was isolated using Trizol^®^ reagent according to manufacturer’s recommendations. Medium free cell monolayers were treated with Trizol^®^ (1ml / cm^2^) and the cells detached from the flask using a cell scraper. After transferring the mixture to centrifuge tubes (1 ml), and incubation at 30 °C for 5 min, choloroform was added (0.2 ml) and the closed tubes shaken vigorously for 15 s, then opened and incubated at 30 °C for 2–3 min. The samples were centrifuged at 12,000× g (2–8 °C) for 15 min and the top aqueous layer carefully transferred to a fresh centrifuge tube. The RNA was precipitated with isopropanol (0.5 ml/ml of Trizol^®^ reagent) and the precipitate washed twice with ethanol and finally dissolved in DEPC- treated water. Primers were designed using Primer 3 Input (Version 0.4.0) and cross-checked using nucleotide BLAST and were: *NPR-A* forward: 5′-GCA TTG AGC TGA CAC GAA AA-3′; reverse: 5′-GTC CAG GGT GAT GCT CTC AT-3′; *NPR-B* forward: 5′-GGC ACA GGA ATC ACC TTC AT-3′; reverse: 5′-GGT GTT GGC AAA GAT CTG GT-3′. The RT–PCR kit reaction mixture consisted of the following: RNA sample 1 µg, PCR buffer (2 reaction mix from the kit) 25 µl, forward primer 2 µl (10 µM stock), reverse primer 2 µl (10 µM stock), reverse transcriptase with Taq polymerase 2 µl, and DNase-free water to make total volume 50 µl. The reaction was run for 30 cycles according to manufacturer’s recommendation and using annealing temperature (Ta) of 55 °C. cDNA synthesis was performed by incubation at 50 °C for 15 min followed by 94 °C for 2 min. The cycling temperatures were as follows: Step 2: 94 °C for 30 s, Step 3: Ta for 30 s, Step 4: 72 °C for 1 min, Step 5: Repeat Steps 2–4 for 30 cycles, Step 6: 72 °C for 5 min, Step 7: 4 °C storage until needed.

### Agarose gel electrophoresis

Agarose gel (1.2%) was prepared by heating agarose in TAE buffer. After cooling, ethidium bromide (6 μl in 100 ml of solution) was added to the mixture and the gels cast in a Horizon 58 (Life Technologies, Carlsbad, CA) apparatus. The total sample obtained from PCR was loaded on to the gel with 5 μl of bromophenol blue dye and 100 bp ladder in two lanes flanking the samples. Electrophoresis was performed at 100 V until the dye reached half the length of the gel.

### Indirect immunofluorescence

#### Corneal tissue immunohistochemistry

Donor corneas were fixed in 4% formaldehyde (4 °C, 24 h), dehydrated through a series of ethanol and xylene incubations, and embedded in paraffin. Embedded tissue was sectioned and the paraffin removed from the sections (~10 µm) by incubations in xylenes and ethanols. After re-hydration (30 min) in PBS (0.256 g/l NaH_2_PO_4_ H_2_O, 1.19 g/l Na_2_HPO_4_, 8.76 g/l NaCl, pH 7.4), and distilled water washes (3×5 min), the tissue sections were blocked overnight at 4 °C in PBS + 1% BSA +1% horse serum. The tissue sections were then rinsed with PBS and distilled water (3×5 min), and incubated with primary (1°) antibody at 4 °C, overnight and rinsed in PBS (3×10 min) containing Tween-20 (0.1%). The tissue sections were then incubated with secondary (2°) antibody (1 h, at room temperature) and rinsed in PBS containing Tween-20 (0.1%, 3×10 min). The specimens were rinsed in PBS (3×10 min), distilled water (3×10 min), stained with 4’,6-diamino-2-phenylindole (DAPI, 220 nM, 10 min) and were mounted using FluorSave™ (Calbiochem, La Jolla, CA).

#### Cellular immunocytochemistry

Approximately 15,000 cells were plated on glass coverslips and cultured in EpiLife^®^ (Cascade Biologics Invitrogen). When the cultures had stabilized, the coverslips were rinsed in PBS and fixed in neutral formalin (4% in PBS, overnight at 4 °C). After re-hydration in phosphate buffered saline (PBS, 0.256 g/l NaH_2_PO_4_ H_2_O, 1.19 g/l Na_2_HPO_4_, 8.76 g/l NaCl, pH 7.4; for 30 min) and distilled water washes (3×), the cells were blocked (overnight at 4 °C) in PBS + 1% BSA (BSA). The cells were then rinsed with PBS and distilled water (3×) and incubated with 1° antibody diluted in PBS at 4 °C overnight. After rinsing in PBS, containing Tween-20 (0.1%; 3×10 min), cells were incubated with 2° antibody at room temperature (RT, 1.5 h) and rinsed in PBS + Tween-20 (0.1%, 3×10 min). The specimens were rinsed in PBS (3×10 min), distilled water (30 min), stained with DAPI (200nm, 10 min) and mounted on glass slides (FluorSave™).

### Antibodies and image acquisition

Primary antibodies for NPR-A and NPR-B were used at 1:100 dilution. Conjugated Alexa Fluor 594 nm goat anti-rabbit was used as the secondary antibody at a concentration of 6 mg/ml of 1% BSA; secondary antibodies were used at dilutions of 1:1,000. Negative controls in all experiments were specimens labeled with 2° antibody only and DAPI to show nuclei. Mounted specimens were examined on Olympus AX70 (Olympus America, Inc., Center Valley, PA,) fluorescent microscope using SPOT Twain software (Microsoft, Issaquah, WA).

### Western blot analysis

Cells were cultured as described above and when near confluent were washed with PBS and treated with lysis buffer (300 μl, 2.5 ml of 1 M TRIS buffer [pH 7.0], 1 g SDS, 2.5 g sucrose in 50 ml of distilled water) for 5 min at room temperature. The genomic DNA was sheared by several passes through a 22 gauge needle and samples were stored at −20 °C until needed. BCA (bicinchoninic acid) protein assays (Pierce, Rockford, IL) were performed to determine protein concentrations and to ensure equal loading of lanes. Protein lysates were mixed with 3 μl of loading buffer and heat denatured for 5 min. Protein (30 μg) was loaded in each lane of the denaturing SDS–PAGE (12% separating and 4% stacking), which was run at 150 V with Tris/glycine as the running buffer. Protein bands were transferred on to the nitrocellulose membrane (VWR International, Irving, TX) by electro-blotting overnight (4 °C) at 10 V in Tris/Glycine buffer with 20% methanol and confirming the transfer with Ponceau Red of the membranes. After de-staining in distilled water the membranes were incubated in blocking buffer (5% powdered milk and 1% BSA in PBS) for 3 h at room temperature. Membranes were then incubated for 30 min (RT), then overnight (4 °C), and finally the following morning for 30 min (RT) with primary antibodies against NPR-A and NPR-B diluted to 1:1,500 and 1:3,000, respectively, in PBS containing 0.1% Tween-20 (PBST) on a plate rocker. After rinsing in PBST containing 0.1% Tween-20 (3×10 min), the membranes were incubated with 2° antibody (anti rabbit IgG from donkey HRP conjugated) dissolved at a dilution of 1:1,000 in PBST, for 1.5 h (RT). After washing with PBST 3×10 min, the membranes were developed by ECL (GE Healthcare, Piscataway, NJ) chemiluminescence.

### Measurement of GC activity in cultured cells

Cells were seeded in 48 wells plates and upon reaching confluence they were rinsed twice with 0.5 ml Dulbeco’s modified Eagle’s medium (DMEM)/F-12. Cells were then pre-incubated for 20 min in the presence or absence of both NPR-A or NPR-B antagonists in DMEM/F-12 containing 1.0 mM of the phosphodiesterase inhibitor 3-isobutyl-1-methylxanthine (IBMX) at 23 °C. NPs were added at the end of this period, and the reaction was allowed to proceed for another 15 min at 23 °C. After aspiration of the medium, ice-cold 0.1 M acetic acid (150 μl, pH 3.5) was added to each well for the termination of cGMP synthesis and cell lysis. Finally, ice-cold 0.1 M sodium acetate (220 μl, pH 11.5–12.0) was added to neutralize the samples before analysis of cGMP produced by an enzyme immunoassay kit (Amersham, Piscataway, NJ) as recommended by the manufacturer and as previously described for cAMP and cGMP quantification in various tissues/cell-types [20,29-31].

## Results

### RT–PCR

**Figure 1:**
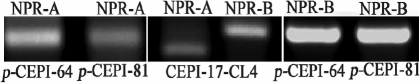
Detection of *NPR-A* and *NPR-B* mRNAs in human *p*-CEPI and CEPI-17-CL4 cells by RT–PCR. Lanes 1 and 2: *NPR-A* in human *p*-CEPI cells (donor ages 64 and 81); Lane 3: *NPR-A* in human CEPI-17-CL4 cells. The PCR product for *NPR-A* is equal to 179 bp. Lane 4: *NPR-B* in human CEPI-17-CL4 cells. The PCR product for *NPR-B* is equal to 237 bp. Lanes 5 and 6: *NPR-B* in human *p*-CEPI cells (donor ages 64 and 81).

mRNAs for *NPR-A* and *NPR-B* were shown to be present in both *p*-CEPI and CEPI-17-CL4 cells (Figure 1).

### Immunohisto- and immunocyto- chemistry

**Figure 2:**
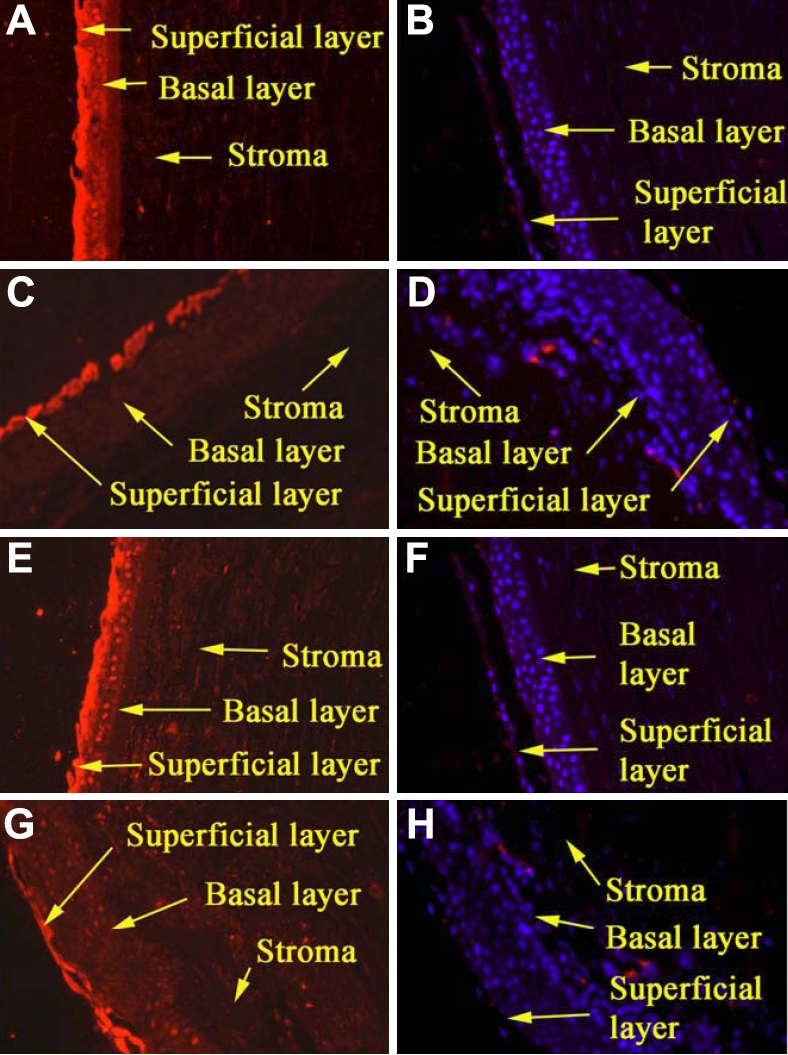
Expression of NPR-A and NPR-B in paraffin sections of formalin-fixed human corneas as determined by indirect immunofluorescence. **A**, **B**: The presence of NPR-A in the epithelium of the central cornea. **A** shows stronger expression in the superficial layers when compared with the control (**B**). **C**, **D**: **C** shows the presence of NPR-A primarily in the superficial layers of the limbal epithelium. The control shows no expression (**D**). **E**, **F**: **E** shows strong expression of NPR-B primarily in the superficial cell layers of the central cornea compared when compared with the control (**F**). **G**, **H**: **G** shows that NPR-B is very distinctly confined to the superficial epithelium of the limbus compared with the control (**H**). All panels are at 10× magnification.

**Figure 3:**
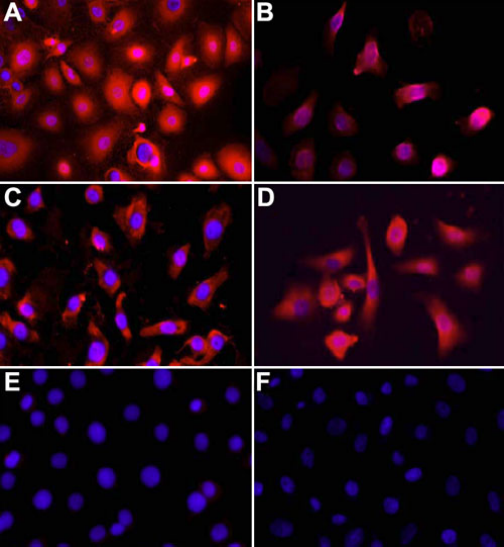
The expression of NPR-A and NPR-B in formalin-fixed human *p*-CEPI and CEPI-17-CL4 cells by indirect immunofluorescence is shown. **A** shows a strong expression of NPR-A in human *p*-CEPI cells, while **B** shows expression of NPR-A in CEPI-17-CL4 cells. **C** shows expression of NPR-B in human *p*-CEPI cells while **D** shows expression of NPR-B in CEPI-17-CL4 cells. **E** and **F** are DAPI labeled controls (treated with secondary antibody only) for human *p*-CEPI and CEPI-17-CL4 cells, respectively. Each panel is at 40× magnification.

Expression of NPR-A and NPR-B was observed in the epithelium of human corneas and was particularly strong in the superficial layers of the central region (Figure 2A-D). NPR-B appeared to be expressed more strongly than NPR-A in the epithelium of the limbus and the expression of both was confined to the superficial layers. Similarly, immunocytochemical analysis of the cultured cell monolayers showed what appeared be cytoplasmic expression of both NPR-A and NPR-B (Figure 3).

### Western blot analysis

**Figure 4:**
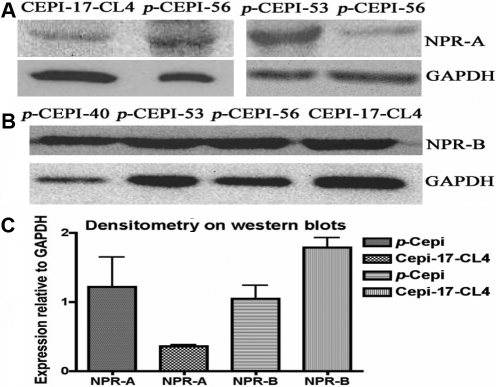
Expression of NPR-A and NPR-B in human *p*-CEPI cells and CEPI-17-CL4 cells as determined by western blot analysis. **A**: The presence of NPR-A protein (band observed at 40–55 kDa) in CEPI-17-CL4 and in human *p-*CEPI cells (from 56, 53, and 56 year old donors) is shown. **B**: The presence of NPR-B protein (band observed at 24 kDa) is shown for three different donors of human *p-*CEPI cells (ages 40, 53, and 56) cells and in CEPI-17-CL4 cells. **C**: The expression of NPR-A and NPR-B in human *p*-CEPI and CEPI-17-CL4 cells was normalized to GAPDH. The figure shows an apparent lower expression of NPR-A in CEPI-17-CL4 cells than in human *p*-CEPI cells (p<0.1); the expression of NPR-B was higher in CEPI-17-CL4 than that in human *p*-CEPI cells (p<0.05). The NPR-B expression was greater than NPR-A expression in CEPI-17-CL4 cells (p<0.05). However, the both receptor subtypes were expressed to the same extent in the *p*-CEPI cells (p<0.1).

Cellular NPR-A and NPR-B protein expression was confirmed by western blot analysis of *p*-CEPI and CEPI-17-CL4 cell lysates (Figure 4A,B). Some donor variability was observed in the levels expressed by *p*-CEPI cells. After normalization of expression to the house-keeping enzyme gene (glycerol-3-phosphate dehydrogenase; GAPDH), CEPI-17-CL4 cells were shown to have a higher expression of NPR-B than *p*-CEPI cells (p<0.05), while the latter cells had a higher expression of NPR-A than CEPI-17-CL4 cells though this did not reach statistical significance (p<0.1; Figure 4C). In addition, CEPI-17-CL4 cells expressed a greater amount of NPR-B than the *p*-CEPI cells (p<0.05) but this could have been a reflection of greater variability in the isolated cells from human donor eyes.

### Pharmacological characterization of NPRs in CEPI Cells

**Figure 5:**
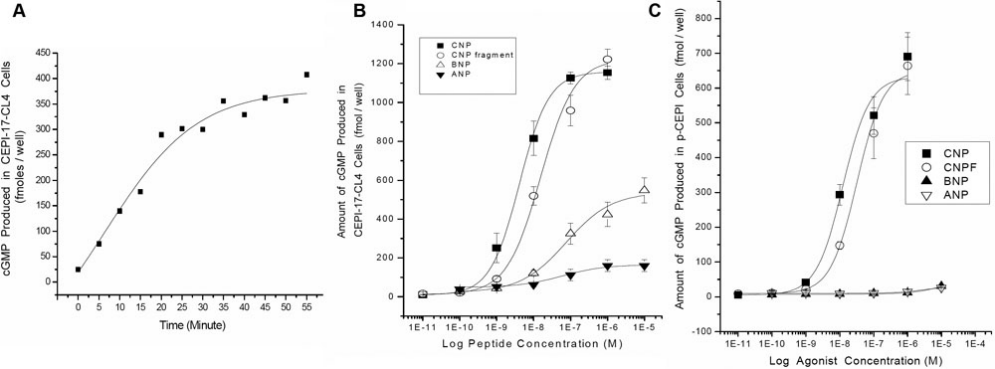
**A**: Time-course of increase in cGMP induced by CNP fragment in CEPI-17-CL4 cells is shown. Cyclic GMP concentration was measured after the cells were treated with CNP fragment for the indicated period. Each symbol represents a single datum point determined in duplicate. **B** and **C**: Effects of NPs on cGMP production in human *p-*CEPI and CEPI-17-CL4 cells are shown. Cyclic GMP concentration was measured after the cells were treated with the indicated concentrations of various NPs for 15 min. Each symbol represents a single datum point determined in duplicate. Results were obtained in 4 independent studies for CEPI-17-CL4 cells (**B**) and 3 independent studies for human *p*-CEPI cells (**C**).

In the presence of IBMX, activation of GC in the ocular cells by NPs resulted in cGMP accumulation. Time-course studies with CNP fragment (1 μM) revealed that for up to 25 min there was a linear increase of cGMP production with a good signal-to-noise ratio (Figure 5A). For convenience, a 15 min stimulation time point was then applied to all other pharmacological studies.

CNP, CNP fragment, BNP, and ANP stimulated the production of cGMP in CEPI-17-CL4 cells in a concentration-dependent manner with the first two peptides causing the highest accumulation of cGMP (Figure 5B; Table 1). ANP and BNP behaved as partial agonists relative to CNP and the CNP fragment. The basal level of cGMP in cell lysates was 24±5 fmol/well (mean±SEM, n=24). Incubation of the cells with 10 μM ANP for 15 min increased the accumulation of cGMP sevenfold to 160±31 fmol/well (n=4). The concentration-response curves for ANP yielded apparent potency (EC_50_) of 97±25 nM (n=4) in these cells. The stimulatory effect of BNP was 23 fold above the basal level, with EC_50_s of 203±95 nM (n=4). In contrast, NPR-B receptor subtype-selective agonist CNP, increased the accumulation of cGMP 53 fold above the basal level to 1,271±133 fmol/well (n=6) with EC_50_s of 7±2 nM (n=8). A 22-amino acid fragment of CNP (CNP-F) increased the level of cGMP 47 fold above the basal level to 1,135±96 fmol/well (n=5) with EC_50_s of 16±2 nM (n=5). The various NPs tested were all significantly more potent (p<0.01) in the CEPI-17-CL4 cells than in the *p*-CEPI cells (Table 1).

**Table 1:** Relative potencies and intrinsic activities of various NPs for their ability to stimulate cGMP production in human CEPI cells compared with other human ocular cell-types.

**Natriuretic peptide**	**Potency (nM) and intrinsic activity (E_max_) in *p*-CEPI Cells**	**Potency (nM) and intrinsic activity (E_max_) in CEPI-17-CL4 Cells**	**Potency in h-CM cells (nM)#**	**Potency in h-TM16 cells (nM)#**	**Potency in h-TM3 cells (nM)#**
**CNP (1–53 amino acids)**					
EC_50_ (nM)	24 ± 5 nM	7 ± 2 nM**	19 nM	10 nM	64 nM
Max. effect (%)	100%	100%			
**CNP fragment (32–53 amino acids)**					
EC_50_ (nM)	51 ± 8 nM	16 ± 2 nM**	nd	nd	nd
Max. effect (%)	96%	89%			
**BNP (32 amino acids)**					
EC_50_ (nM)	>10,000 nM	203 ± 95 nM**	35 nM	75 nM	398 nM
Max. effect (%)	4.5%	43%			
**ANP (1–28 amino acids)**					
EC_50_ (nM)	>10,000 nM	97 ± 25 nM**	102 nM	102 nM	478 nM
Max. effect (%)	3.8%	13%			

Concentration-response curves were generated for each of the NPs shown above using 6 concentrations of each peptide in each experiment as shown in Figure 5B,C. Data shown above are mean±SEM from 3 to 4 experiments for human *p*-CEPI cells and from 4 to 9 experiments for CEPI-17-CL4 cells. #=data from [18] for comparison. h-CM=human ciliary muscle cells; h-TM=human trabecular meshwork cells; nd=not determined. **=p<0.01 relative to potency in *p*-CEPI cells by Student’s unpaired *t*-test.

NPs were also effective in increasing cGMP accumulation in *p*-CEPI, and as in the CEPI-17-CL4 cells, the most efficacious and potent NPs were CNP and CNP fragment (Figure 5C). The basal level of cGMP in *p*-CEPI cells was 7.8±1.5 (mean±SEM, n=10). Treatment of the *p*-CEPI cells with 10 µM ANP increased the cGMP level threefold above basal to 26±1 fmol/well (n=3). BNP was not as potent as ANP and its maximal stimulation resulted in 31±1.5 fmol/well (n=3) of cGMP. The EC_50_ of both ANP and BNP peptides was >10,000 nM. Again, in contrast with ANP and BNP and similar to it’s effect in CEPI-17-CL4 cells, CNP, the NPR-B selective agonist was much more potent than ANP with the mean EC_50_s of 24±5 nM (n=4) and 27 times more efficacious than ANP yielding cGMP levels of 690±68 fmol/well (n=3). CNP fragment was as efficacious as CNP and it increased the accumulation of cGMP to 664±83 fmol/well (n=3) with EC_50_s of 51±8 nM. As for CEPI-17-CL4 cells above, the pharmacological profiles of these compounds suggested that NPR-B was also the most prominent subtype of NPR in *p*-CEPI cells (Figure 5C; Table 1).

**Figure 6:**
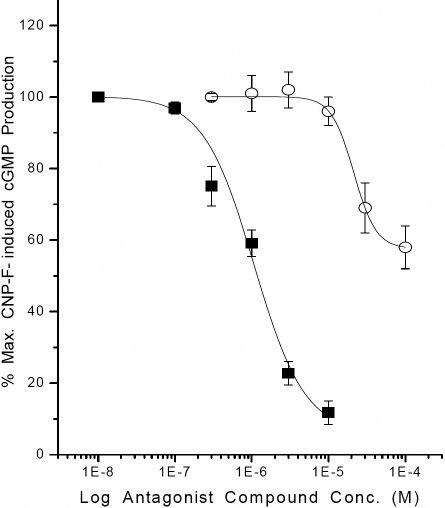
Inhibition of CNP fragment-induced cGMP production in CEPI-17-CL4 cells by two NP receptor antagonists is shown. The effects of HS-142–1 (10 nM to 10 µM; closed square symbols) and isatin (0.3–100 µM; open circle symbols) on CNP fragment (30 nM)-induced cGMP generation were determined as described in the methods section. Data are mean±SEM from 6 to 7 experiments for HS-142–1, and from 7 experiments for isatin. Similar results were obtained using *p*-CEPI cells. The antagonist potencies of the two compounds in the two cell-types are provided in the Results section.

To further characterize the pharmacological profile of the NPRs in the CEPI cells, HS-142–1 was evaluated for its antagonist activity. HS-142–1 inhibited CNP-induced cGMP production in a concentration-dependent manner with the inhibitory potency (K_i_) of 0.25±0.05 µM (n=6) in CEPI-17-CL4 (Figure 6), and with a K_i_ value of 0.44±0.09 µM (n=7) in *p*-CEPI cells. HS-142–1 was thus equieffective in both cell types (p<0.1). Isatin (indole-2,3 dione), an NPR-A receptor antagonist, partially inhibited CNP-induced cGMP production, and was less potent (K_i_=5.3±1.7 µM; n=7 in CEPI-17-CL4, and K_i_=7.8±4.5 µM; n=3 in *p*-CEPI cells) than HS-142–1 (Figure 6).

## Discussion

As described in the Introduction, diseases or injuries of the corneal epithelium compromise its functions and can, if left untreated, result in loss of visual acuity and impair vision [21,25]. For these and other reasons, we and others have been involved in the study of ocular surface tissues and cells [21,25-28,30,32-39].

Various pharmacologically important peptides and proteins have been evaluated in the cornea and it is becoming clear that in spite of its avascularity the corneal epithelium responds to endogenous (from paracrine/ autocrine sources) and exogenous biologically active peptides and cytokines [23,26,27]. The presence of the natriuretic family of peptides and their receptors have not been previously documented in the human cornea. Since some of the functions of this peptide family have been implicated in cell proliferation and differentiation [5-7,24], which are the essential events in the homeostasis and repair of the corneal epithelium [40], we undertook the present study to demonstrate that their presence might also be of physiologic and pharmacological significance. We first showed the presence of mRNAs for *NPR-A* and *NPR-B* indicating that in monolayer cellular model of corneal epithelium (*p*-CEPI and CEPI-17-CL4 cells) the genes are actively involved in the transcription process in the cornea and that the cell-line CEPI-17-CL4 could be a good model for in vitro cellular studies. Examination of tissue sections of the human cornea showed that the epithelium is a target for the NPs and that the NPR expression favors the superficial epithelial cell layers, particularly in the limbus. This seems reasonable since the tear film compartment is known to harbor a variety of bioactive peptides [25,27,41] and the ocular surface includes the conjunctiva, which is a particular site for allergic and inflammatory reactions [26,27,36,37,41-43]. The presence of NPR-A and NPR-B was also shown by immunocytochemistry in *p*-CEPI and CEPI-17-CL4 cells and confirmed by western blot protein analysis. Although there was some donor variability in the expression of NPR-A and NPR-B by *p*-CEPI cells, it was interesting to observe that while *p*-CEPI cells expressed higher levels of NPR-A, CEPI-17-CL4 cells favored NPR-B expression. This difference suggests that as a prelude to in vivo studies it is worthwhile to be aware of the possible differences in responses between cell-line cells and primary cells [26]. Thus, as it is demonstrated here, it is prudent to include primary human cells in a multi-disciplinary approach to studies of pharmacologically active agents [25-28]. Irrespective of the immunocytochemical observations, however, the pharmacological data coupled with the [cGMP]_i_ production-response data indicated that the functionally active subtype of the NPRs was the NPR-B in both the *p*-CEPI and CEPI-17-CL4 cells. The apparent lower expression level of NPR-A may have accounted for such a difference in CEPI-17-CL4 cells (Figure 4B), however NPR-B still functionally predominated in the *p*-CEPI cells where the expression levels of NPR-A and NPR-B were very similar (Figure 4B). Another interesting difference between the cell-types was the fact that the various NPs studied currently were more potent in the immortalized cells than in the *p*-CEPI cells (Table 1; Figure 5B,C). This could be explained by the relative homogeneity of the cell-line cell population, as compared the heterogeneity of the isolated primary cells from various human donor eyes.

Exposure of *p*-CEPI and CEPI-17-CL4 cells to various NPs resulted in a concentration-dependent increase in [cGMP]_i_ indicating that these cells have the ability to translate the mRNAs for NPRs into functionally active receptor proteins. These results further supported the molecular biologic and immunocytochemical data discussed above. In view of the fact that CNP and CNP fragment exhibited the highest potencies and intrinsic activities in elevating [cGMP]_i_ in *p*-CEPI and CEPI-17-CL4 cells, with both ANP and BNP being much weaker, these findings indicated an NPR-B pharmacology signature as previously documented for cells derived from other tissues, including human TM and CM cells [18]. Interestingly, the rank order and potency values of CNP, BNP, and ANP in the current studies matched well with those reported for h-CM and h-TM cells (Table 1) [18]. The fact that the agonist activity of CNP fragment was relatively potently blocked by the NPR-B selective antagonist, HS-142–1 [44-46], and weakly by isatin [47-49], an NPR-A antagonist, confirmed the identity of the predominantly active GC-activating NPR in *p*-CEPI and CEPI-17-CL4 cell-types as the NPR-B sub-type. Additionally, the inhibitory potency of HS-142–1 in these cells (K_i_=0.25–0.44 µM) for blocking the CNP fragment-induced [cGMP]_i_ generation compared well with its reported antagonist potency in numerous other organ-derived cells-types (K_i_=0.33–6.6 µM) [44-46]. Even though isatin yielded a partial inhibition of CNP fragment-induced [cGMP]_i_ production in the CEPI cells, its potency was similar to that previously reported for its inhibitory activity in non-ocular cell-types (K_i_=0.4 µM) [47-49]. Taken together, the pharmacological profile of the NP agonist and antagonist activities confirmed the predominance of a functionally active NPR-B receptor sub-type in *p*-CEPI and CEPI-17-CL4 cells.

Even though it is now clear from our current studies that CNP-sensitive NPR-B receptors are present on both *p*-CEPI and CEPI-17-CL4 cells and in the intact human corneal epithelium, the potential physiologic relevance of these findings have not been fully explored and warrant future investigation. However, the fact that NPs are potent regulatory mediators of secretory activity [5-7,50] in many epithelial cell-types of the body, for instance leading to chloride secretion [50], suggests that CNP may modulate electrolyte composition of the tear film on the ocular surface by influencing the activity of the NPR-B on the CEPI cells. Since CNP upregulates aquaporin-4 [51], the water content of the tear film may be directly regulated by corneal NPR-B receptors. These combined effects of CNP on the corneal cells in vivo may then regulate water and ion homeostasis [51,52] on the ocular surface to maintain a stable environment for the cornea and surrounding tissues. Furthermore, since CNP inhibits leukocyte recruitment and platelet-leukocyte interactions [53], the NPR-B receptors may participate in anti-inflammatory effects of CNP on the ocular surface [53]. The involvement of NP system in cell proliferation [4-7,24] necessary for corneal wound healing [40] is also possible even though ANP apparently inhibited cell proliferation induced by epidermal growth factor (EGF) via the NPR-A subtype [24]. Unfortunately, the latter authors did not study the effects of CNP on rabbit CEPI proliferation and thus the role of NPR-B in this process remains undefined [24]. Our preliminary studies have revealed that CNP fragment neither alone or in combination with EGF or HS-142–1 influences CEPI cell proliferation (data not shown) indicating that in human CEPI cells NPR-B appear not to be involved in modulating cellular growth.

Corneal epithelial cells, akin to other ocular epithelia [52], express numerous ion-channels and a multitude of receptors [25-28]. Since CNP increases cGMP via NPR-B, as does carbon monoxide but via a soluble GC [54], and CNP alters intracellular pH [55], the activation of K^+^-channels [54] and inhibition of Na-K-ATPase [56] of the CEPI cells may be also affected. The solitary or combined effects of CNP’s activation of NPR-B to produce [cGMP]_i_ may thus have profound effects on the physiologic functions of the corneal epithelial cells [40,50-57], including possible homeostatic regulation of ocular surface osmolarity and/or cell volume, and/or acting as mitochondrial protective agents as it pertains to other ocular tissues [58].

In conclusion, a multidisciplinary approach allowed us to probe the molecular biology and biochemical pharmacology of the NPR system in the human corneal epithelium at the cellular and tissue levels. Compelling data are presented here that indicate the predominance of NPR-B whose activation leads to the generation of [cGMP]_i._ The agonist profile of activity of NPs in *p*-CEPI and CEPI-17-CL4 cells, coupled with the antagonist profile obtained using HS-142–1 and isatin, supported the immunohistochemical findings. It is hoped that our collective data will stimulate further investigations into determining the physiologic roles of the NPR system in the human and animal corneal epithelium.
